# Inhibiting SUMO1-mediated SUMOylation induces autophagy-mediated cancer cell death and reduces tumour cell invasion via RAC1

**DOI:** 10.1242/jcs.234120

**Published:** 2019-10-15

**Authors:** Mar Lorente, Ana García-Casas, Nélida Salvador, Angélica Martínez-López, Estibaliz Gabicagogeascoa, Guillermo Velasco, Lucía López-Palomar, Sonia Castillo-Lluva

**Affiliations:** 1 Departamento de Bioquímica y Biología Molecular, Facultad de Ciencias Químicas y Biológicas, Universidad Complutense, Madrid 28040, Spain; 2 Instituto de Investigaciones Sanitarias San Carlos (IdISSC), Madrid 28040, Spain; 3 Instituto Universitario de Investigación Neuroquímica, Universidad Complutense, Madrid 28040, Spain

**Keywords:** SUMO modification, Rho-GTPase, Breast cancer, Autophagy, Ginkgolic acid, TRIB3

## Abstract

Post-translational modifications directly control protein activity and, thus, they represent an important means to regulate the responses of cells to different stimuli. Protein SUMOylation has recently been recognised as one such modification, and it has been associated with various diseases, including different types of cancer. However, the precise way that changes in SUMOylation influence the tumorigenic properties of cells remains to be fully clarified. Here, we show that blocking the SUMO pathway by depleting SUMO1 and UBC9, or by exposure to ginkgolic acid C15:1 or 2-D08 (two different SUMOylation inhibitors), induces cell death, also inhibiting the invasiveness of tumour cells. Indeed, diminishing the formation of SUMO1 complexes induces autophagy-mediated cancer cell death through increasing the expression of Tribbles pseudokinase 3 (TRIB3). Moreover, we found that blocking the SUMO pathway inhibits tumour cell invasion by decreasing RAC1 SUMOylation. These findings shed new light on the mechanisms by which SUMO1 modifications regulate the survival, and the migratory and invasive capacity of tumour cells, potentially establishing the bases to develop novel anti-cancer treatments based on the inhibition of SUMOylation.

## Abstract (precis)

**Summary:** Blockade of the SUMO pathway inhibits tumorigenesis through two different mechanisms: (i) stimulation of autophagy-mediated cancer cell death via TRIB3 upregulation; and (ii) inhibition of invasiveness via abrogation of RAC1 SUMOylation.

## INTRODUCTION

The post-translational addition of the small ubiquitin-related modifier (SUMO) peptide is now established as one of the key regulatory modifications in eukaryotic cells. SUMOylation involves the reversible binding of a SUMO peptide to a lysine residue in the target protein and, to date, four different SUMO isoforms have been identified: SUMO1, SUMO2 and SUMO3 (denoted SUMO2/3 because they have a high degree of similarity), and SUMO4 (Saitoh and Hinchey, 2000). The addition of these peptides is mediated by an enzyme cascade that includes an activating enzyme (heterodimer SAE1/2), an E2-conjugating enzyme (UBC9) and an E3 ligase (RANBP2 from the SIZ/PIAS family and members of the ZNF451 family) (Hay, 2005; Pichler et al., 2017), and the substrates can be modified by adding a single SUMO moiety, multiple SUMOs or SUMO chains (Pichler et al., 2017). In addition, specific SUMO-modified proteins can be deSUMOylated by a group of sentrin/SUMO-specific proteases (SENPs) (Hay, 2007).

The conjugation of a SUMO moiety affects proteins distinctly, modifying their activity, subcellular localization or stability. Many cellular pathways are influenced by reversible SUMOylation, such as those that influence chromatin organization, transcription, DNA repair, macromolecular assembly, protein homeostasis, trafficking and signal transduction (Geiss-Friedlander and Melchior, 2007). Failure to SUMOylate specific targets and the global misregulation of SUMOylation has been linked to different diseases, including cancer and heart failure (Flotho and Melchior, 2013). Therefore, interfering with the SUMOylation machinery could represent a novel therapeutic approach in the management of some diseases.

Ginkgolic acids (GAs) are a group of alkyl phenols found in crude extracts of *Ginkgo biloba* leaves, an ancient gymnosperm species now distributed globally (Mahadevan and Park, 2008). There are several molecular species of GA; these have a different length for their alkyl group within the main structure of the molecule (C13:0, C15:1 and C17:1). GAs display anti-cancer activity, and in several studies GA has been shown to inhibit the growth and invasion of a number of cancer cell types, including pancreatic, liver, pharyngeal and colon cancer (Qiao et al., 2017). While the mode of action of these compounds is still poorly understood, GA C15:1 has been shown to directly bind to E1 activating enzymes and impair the formation of the E1–SUMO1 intermediate (Fukuda et al., 2009). However, it remains to be clarified whether the anti-cancer activity of GAs depends on inhibition of the SUMO machinery or if additional mechanisms are involved in this effect.

RAC1 is a member of the Rho family of small GTPases that act as molecular switches to control a wide array of cellular events. RAC1 activity can modulate the cytoskeleton, which is critical for a number of cellular activities such as phagocytosis, mesenchymal-like migration, axon growth, adhesion, cell differentiation and cell death mediated by reactive oxygen species (ROS) (Acevedo and Gonzalez-Billault, 2018). RAC1 also plays an important role in moderating other signalling pathways that influence cell growth and the cell cycle (Mettouchi et al., 2001; Olson et al., 1995), the formation of cell–cell adhesions (Daugaard et al., 2013) and contact inhibition (Nobes and Hall, 1995). These RAC1-mediated activities appear to be central to the processes that underlie malignant transformation, including tumorigenesis, angiogenesis, invasion and metastasis (Mack et al., 2011).

The RAC1 GTPase binds to either GTP or GDP, the exchange of which controls its activation. RAC1 is inactive in the GDP-bound state and it is activated upon exchange of its GDP for GTP, enabling downstream signalling to proceed. RAC1 activity can be regulated through its association with several guanine nucleotide-exchange factors (GEFs) and GTPase-activating proteins (GAPs), these controlling the cycling between the GDP- and GTP-bound states. Furthermore, post-translational modifications (PTMs) of RAC1 can also regulate its activity. As such, modification of the C-terminal CAAX motif in RAC1 through the addition of either farnesyl or geranylgeranyl isoprenoid lipids increases its hydrophobicity, facilitating both its membrane localization and activation (Mack et al., 2011). Ubiquitin-like (UBL) modifications of RAC1 have also been shown to regulate its activity, including ubiquitylation (Castillo-Lluva et al., 2013) and SUMOylation (Castillo-Lluva et al., 2010), adding further complexity to the regulation of RAC1 signalling.

We observed RAC1 GTPase SUMOylation (RAC1-SUMO1) when the epithelial to mesenchymal transition (EMT) was induced by hepatocyte growth factor (HGF) (Castillo-Lluva et al., 2010). EMT involves changes in gene expression, and it is associated with a loss of cell polarity and an increase in cell invasiveness (Brabletz et al., 2018). The RAC1 GTPase plays an important role in the EMT programme (Ungefroren et al., 2018) and significantly, RAC1 SUMOylation is necessary for optimal cell migration when non-tumorigenic cells undergo EMT. Similarly, cancer cells also induce the EMT programme when they metastasize and invade other tissues (Brabletz et al., 2018), such that RAC1 SUMOylation could also play an important role in this context.

Here, we demonstrate that blockade of the SUMO1 conjugation pathway inhibits two of the cellular programmes that are activated during tumorigenesis, cancer cell survival and invasiveness. These effects are due to the activation of two independent mechanisms: the induction of autophagy-mediated cancer cell death through enhanced TRIB3 expression, and inhibition of RAC1-dependent cancer cell migration and invasion. Tumour cell invasion and metastasis are thought to be responsible for 90% of cancer-associated deaths. Thus, inhibiting SUMOylation could represent a novel therapeutic strategy to convert cancer from a mortal into a chronic disease.

## RESULTS

### Blocking the SUMO pathway inhibits cell viability in breast and prostate cancer cells

**Fig. 1.**
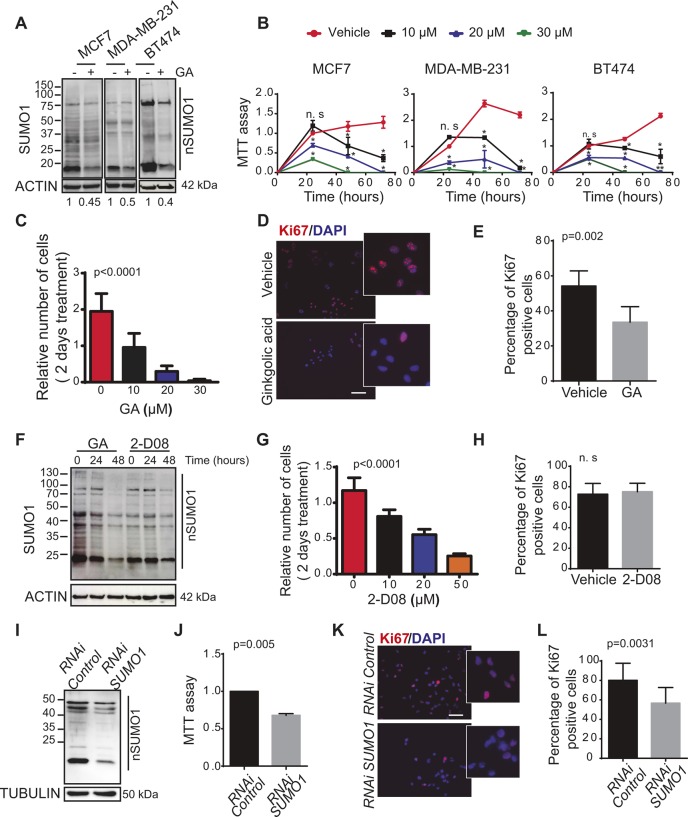
**GA treatment compromises the viability of breast cancer cells.** (A) Expression of SUMO-conjugated proteins (nSUMO1) in different molecular subtypes of breast cancer cells: luminal (MCF7), triple-negative (MDA-MB-231) and HER2+ (BT474) treated with GA (10 µM) for 24 h and analysed by immunoblotting (12% acrylamide gel). (B) MCF7, MDA-MB-231 and BT474 breast cancer cell lines were treated with various concentrations of GA and their cell viability was assessed in an MTT assay: *P≤0.001; **P≤0.0001; n.s., not significant versus non-treated cells (two-way ANOVA). Turkey′s multiple comparisons shows statistical significant differences in all conditions apart from 10 µM GA at 24 h. (C) Number of MDA-MB-231 cells after 2 days of treatment with different concentrations of GA relative to the control (*t*=0 h treatment). (D) Representative image of immunohistochemical evaluation of Ki-67 expression in MDA-MB-231 tumour cells and (E) the percentage of Ki-67-positive cells. (F) Expression of SUMO-conjugated proteins (nSUMO1) in MDA-MB-231 cells exposed to GA (10 µM) or 2-D08 (20 µM) for different times and analysed by immunoblotting (12% acrylamide gel). (G) Number of MDA-MB-231 cells after 2 days of treatment with different concentrations of 2-D08 relative to the controls (*t*=0 h treatment). (H) Percentage of Ki-67-positive MDA-MB-231 cells after 2-D08 treatment for 24 h. (I) SUMO-conjugated proteins in MDA-MB-231 control and SUMO1-depleted cells analysed by immunoblotting (12% acrylamide gel). (J) The viability of MDA-MB-231 SUMO1-depleted cells was evaluated with an MTT assay at 72 h post-depletion. (K) Representative immunofluorescence images of MDA-MB-231 control and SUMO1-depleted tumour cells stained for Ki-67 expression and (L) the percentage of Ki-67-positive cells. All experiments were performed at least three times and the data are the mean±s.e.m. of three independent experiments. Scale bars: 200 μm.

As a first approach to investigate the effect of inhibiting the SUMO pathway on the tumorigenic properties of cancer cells, we analysed the effects of the natural compound GA C15:1 (hereafter referred to as GA), which blocks the SUMO pathway by inhibiting the formation of the E1–SUMO1 intermediate (Fukuda et al., 2009). Exposure of luminal (MCF7), triple-negative (MDA-MB-231) and HER2+ (BT474) breast cancer cells to a relatively low dose of GA (10 µM) reduced the overall protein SUMOylation in these cells by ∼40–50% (Fig. 1A). Moreover, this reduction in SUMO conjugation was more pronounced when these cells were exposed to a higher dose of GA (20 µM, Fig. S1A). When we then tested the effect of this compound on the survival of the different breast and prostate cancer cell lines, exposure to GA clearly diminished their viability in a dose-dependent manner (Fig. 1B; Fig. S1B). Hence, the effect of GA on cell viability appears to be common to different types of cancer cells. We identified the IC_50_ of GA as 10 µM in MDA-MB-231 cells and 20 µM in MCF7 cells (the concentration that reduced the viability of cells by 50% compared with the control after a 2 day treatment; Fig. 1C; Fig. S1C). Accordingly, to study the mechanisms underlying these effects, we used sub-maximal doses of GA (10 µM) to enhance specificity and to avoid any potential toxicity associated with the excessive loss of protein SUMOylation. When we assessed the proliferation of cancer cells through the expression of the Ki67 marker, exposure to GA (10 µM) provoked a decrease in proliferation (Fig. 1D,E) and it reduced the levels of JUN (Fig. S1D), a transcription factor associated with the proliferation of breast cancer cells (Vleugel et al., 2006).

To confirm that the effect of GA observed was due to the inhibition of SUMOylation, we used a different SUMO1 inhibitor (2-D08) that blocks the transfer of SUMO from the E2 enzyme (UBC9) thioester conjugate to the substrate (Choi et al., 2018; Kim et al., 2014). Exposure of the triple-negative cell line (MDA-MB-231) to a relatively low dose of GA and 2-D08 reduced overall protein SUMOylation (Fig. 1F). While GA reduced the intensity of SUMO-conjugated proteins at 24 h, 2-D08 achieved a similar effect after a 48 h exposure. Accordingly, no differences in cell proliferation were observed in the presence of 2-D08 after 24 h, although cell viability was compromised after 48 h in the presence of this SUMO1 inhibitor (Fig. 1G,H).

To further confirm that SUMO1-mediated SUMOylation was responsible for the loss of cell viability observed in the presence of GA, we used a specific pool of siRNAs to deplete SUMO1 over 72 h (Fig. 1I) in the triple-negative MDA-MB-231 breast cancer cell line, a particularly aggressive subtype of breast cancer. As a result, we found that the depletion of SUMO1 reduced cell viability (Fig. 1J) and impaired cell proliferation (Fig. 1K,L).

Together, these results support the notion that inhibition of protein SUMOylation by using genetic or pharmacological approaches reduces the viability and the proliferation of cancer cells.

### Pharmacological inhibition of the SUMO pathway induces autophagy-mediated cancer cell death

**Fig. 2.**
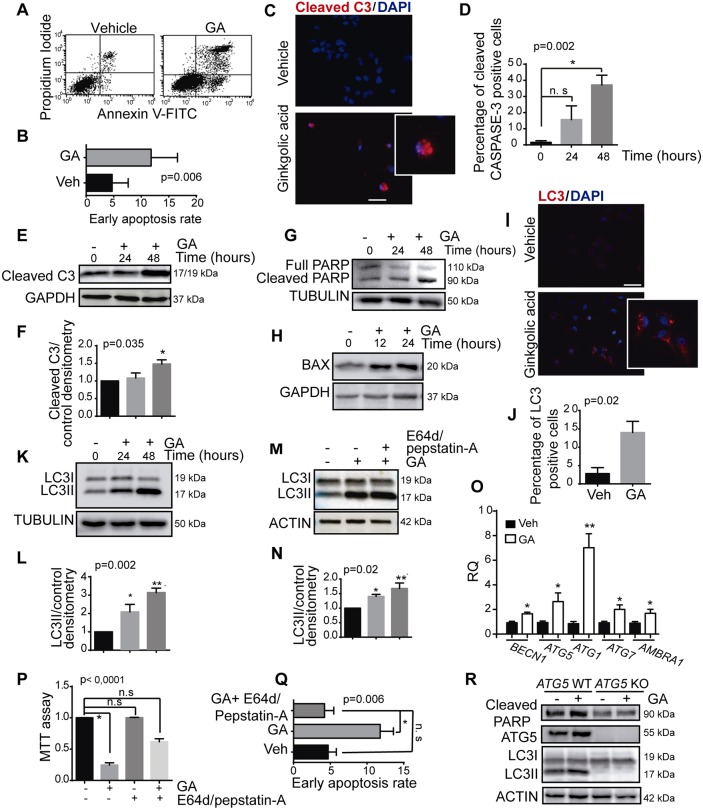
**GA treatment induces autophagy-mediated cell death in breast cancer cells.** (A) Representative flow cytometry results of Annexin V-FITC/propidium iodide (PI) staining showing the level of MDA-MB-231 cell apoptosis after GA treatment for 48 h (0, 10 μM). (B) Quantification of early apoptotic rate measured as the percentage of Annexin V-positive and PI-negative cells (bottom right quadrant in A) (Student's *t*-test). (C) Representative images of cleaved caspase-3 (C3) immunostaining in cells at 48 h and (D) the percentage of active caspase-3-stained cells at different time points. (E) The level of active caspase-3 in MDA-MB-231 cells treated with GA (10 µM) as determined by immunoblotting. (F) Quantification of results in E as determined by densitometry analysis using ImageJ software. (G) The effect of GA on cleaved PARP and (H) BAX at the time points indicated. (I) Representative images of LC3 immunostaining and (J) the proportion of cells with LC3 dots relative to the total number of cells after a 24 h treatment. (K) The effect of GA (10 µM) on LC3 lipidation in MDA-MB-231 cells. (L) Densitometric quantification of results from K using ImageJ software. (M) Effect of GA on LC3 lipidation in MDA-MB-231 cells in the presence of a combination of the lysosomal protease inhibitor E64d and Pepstatin-A at 24 h. (N) Densitometry of results from M for at least three different experiments as quantified with ImageJ software of (ANOVA test and Dunnett's multiple comparisons test). (O) Effect of GA on the expression markers of autophagy as determined by qRT-PCR 12 h after treatment. RQ, relative quantification normalized to vehicle. (P) MDA-MB-231 breast cancer cells were treated with GA (10 µM) and a combination of pepstatin-A and E64d for 48 h, and cell viability was assessed with an MTT assay after 48 h. (Q) Quantification of rate of early MDA-MB-231 cell apoptosis after GA treatment and a combination of Pepstatin-A and E64d for 48 h (ANOVA test and Dunnett's multiple comparisons test). (R) Activation of apoptosis assessed through the level of cleaved PARP in MEF *Atg5-WT* or *Atg5-KO* cells in the presence of GA for 48 h. *P≤0.05, **P≤0.01.; n.s non-significant. All experiments were performed at least three times and the data are the mean±s.e.m. of three independent experiments. Scale bars: 200 μm.

To understand how the SUMO pathway might affect the viability of cancer cells, we analysed the mechanisms activated in response to GA in the triple-negative MDA-MB-231 breast cancer cell line. We measured the rate of MDA-MB-231 apoptosis after a 48 h exposure to GA (10 µM), a time point at which cell viability was compromised (Fig. 1B). By using an Annexin V-FITC and propidium iodide (PI) staining assay, GA was seen to increase the proportion of apoptotic cells after 48 h of treatment (Fig. 2A,B) and indeed, an increase in cleaved caspase-3 and cleaved PARP was also evident in Western blots and by immunofluorescence after a 48 h exposure to GA (Fig. 2C–G). Similar results were also obtained with the SUMO inhibitor 2-D08 (Fig. S2A). Furthermore, we identified an increase in the apoptotic protein BAX at early time points (12 and 24 h, Fig. 2H), suggesting that early activation of the intrinsic mitochondrial pathway might be involved in the stimulation of apoptosis by GA.

We then investigated the mechanisms that might trigger this apoptosis at earlier time points, as other compounds have already been seen to promote apoptotic cancer cell death by stimulating autophagy (Fulda and Kögel, 2015; Salazar et al., 2009b). Autophagy is an essential homeostatic process that facilitates the transport of cellular components to the lysosomal degradation pathway. Depending on the cell context, stimulating autophagy can produce protective or cytotoxic effects (Wilde et al., 2018). We assessed whether the stimulation of autophagy might reduce tumour cell viability in a manner similar to that observed upon SUMO inhibition with GA. A 24 h exposure to GA triggered the accumulation of LC3II (the lipidated and autophagosome-associated form of LC3 family proteins), as assessed by western blotting, in both breast and prostate cancer cells, and as also evident in immunofluorescence analyses (Fig. 2I–L; Fig. S2B–D). At this time point, no change in cell viability had yet been observed. Similar results were also obtained with the SUMO inhibitor 2-D08 (Fig. S2E–G).

It is known that LC3 lipidation (to give LC3II) and its accumulation can be due to both the induction and blockade of autophagy (Klionsky et al., 2016). Pharmacological inhibition of the final step of autophagosome degradation (using E64d and pepstatin-A) led to the accumulation of more LC3II, indicating that GA activated autophagy rather than blocking it (Fig. 2M,N). Furthermore, shorter exposures to GA or 2-D08 induced the expression of regulators of autophagy, such as BECN1, ATG5, ATG1, ATG7 or AMBRA1 (Fig. 2O; Fig. S2H,I). Importantly, there was no decrease in cell viability when MDA-MB-231 cells were treated with GA, E64d and pepstatin-A (Fig. 2P), and the increase in apoptosis evoked by GA was also prevented (Fig. 2Q). Moreover, genetic depletion of three different key autophagy regulatory genes (*ATG5*, *BECN1* and *AMBRA1*) appeared to rescue the apoptosis of these cells upon exposure to GA or 2-D08 (Fig. 2R; Fig. S2J–L).

Together, these observations support the idea that inhibiting SUMOylation induces autophagy-dependent cell death in breast cancer cell lines.

### Pharmacological inhibition of the SUMO pathway induces the upregulation of the autophagy modulator TRIB3

**Fig. 3.**
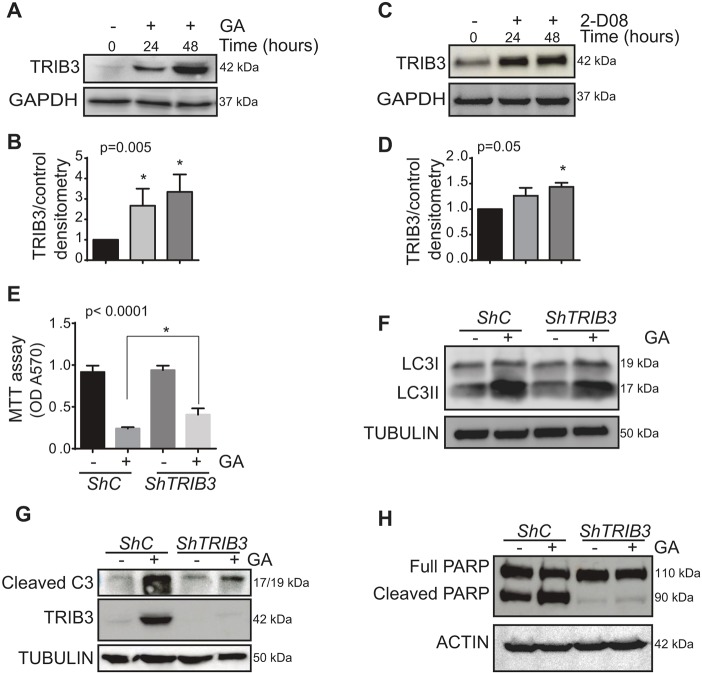
**GA upregulates TRIB3 expression.** (A) MDA-MB-231 cells were treated with GA (10 µM) at different time points and the TRIB3 protein was then analysed by immunoblotting. (B) Densitometry of results in A as determined with ImageJ software. (C) MDA-MB-231 cells were treated with 2-D08 (20 µM) and the levels of the TRIB3 protein was analysed at different time points by immunoblotting. (D) Densitometry of results in C as determined the ImageJ software. (E) MDA-MB-231 control (*ShC*) and TRIB3-depleted (*ShTRIB3*) cells were treated for 48 h with GA (10 µM) and cell viability was determined with an MTT assay (ANOVA test and Dunnett's multiple comparisons test). (F) LC3 lipidation in MDA-MB-231 control and TRIB3-depleted cells treated for 24 h with GA was assessed by immunoblotting. (G) Activation of apoptosis as determined through assessment of the level of active caspase-3 or (H) cleaved PARP in immunoblots of MDA-MB-231 control and TRIB3-depleted cells treated for 48 h with GA. All the experiments were performed at least three times and the data are the mean±s.e.m. of three independent experiments. *P≤0.05.

Several anti-cancer agents that stimulate autophagy-dependent cell death produce their effects by upregulating Tribbles pseudokinase-3 (TRIB3), including natural compounds of lipid origin that exhibit structural similarities to GA (Erazo et al., 2016; Salazar et al., 2009a; Vara et al., 2011). TRIB3 is an upstream regulator of the mammalian target of rapamycin complex 1 (mTORC1) (Salazar et al., 2015), the inhibition of which is considered to be the main mechanism triggering autophagy in many cell settings (Mizushima et al., 2008). Thus, we asked whether the stimulation of autophagy-mediated cell death through pharmacological blockade of the SUMO pathway might be related to changes in the expression of this protein. Exposure to GA increased the levels of TRIB3 in breast and prostate cancer cell lines (Fig. 3A,B; Fig. S3A), as did exposure to the 2-D08 SUMO inhibitor (Fig. 3C,D). Therefore, we hypothesized that the upregulation of TRIB3 might be the primary event leading to autophagy-mediated cell death in response to pharmacological inhibition of the SUMO pathway. In fact, stable knockdown of *TRIB3* expression in MDA-MB-231 cells rendered these cells more resistant to GA-induced autophagy and cell death (Fig. 3E,F), preventing the accumulation of the cleaved forms of caspase-3 and PARP (two well established read-outs of apoptosis) (Fig. 3G,H).

Hence, these observations led us to conclude that TRIB3 induction is involved in the stimulation of autophagy-mediated apoptotic cell death upon pharmacological inhibition of the SUMO pathway.

### Genetic inhibition of SUMO1, but not of SUMO2/3, induces TRIB3 upregulation and autophagy-mediated cancer cell death

**Fig. 4.**
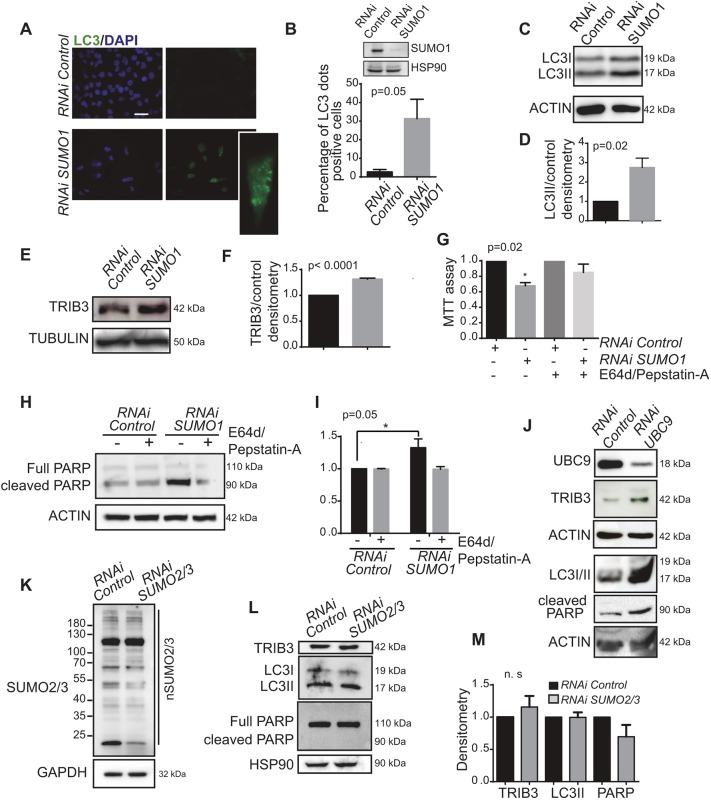
**SUMO1 depletion induces autophagy and TRIB3 expression.** (A) Representative images of LC3 immunostaining and (B) the percentage of cells with LC3 dots relative to the total number of cells. (C) The effect of 72 h SUMO1 depletion on LC3 lipidation in MDA-MB-231 cells. (D) Densitometry quantification of results from C as determined with ImageJ software. (E) TRIB3 protein levels were analysed by immunoblotting of SUMO1-depleted MDA-MB-231 cells. (F) Densitometry quantification of results from E as determined using ImageJ software. (G) MDA-MB-231 control and SUMO1-depleted cells were treated for 24 h with E64d and Pepstatin-A, and cell viability was assessed with an MTT assay. (H) Activation of apoptosis (levels of cleaved PARP) was assessed by immunoblotting in MDA-MB-231 SUMO1-depleted cells in the presence of E64d and Pepstatin-A. (I) Densitometry quantification results from H as determined using ImageJ software. (J) The activation of autophagy (levels of LC3II, TRIB3) and apoptosis (levels of cleaved PARP) was analysed in MDA-MB-231 control and UBC9-depleted cells. (K) Expression of SUMO2/3-conjugated proteins (nSUMO2/3) in MDA-MB-231 control and SUMO2-depleted cells were analysed by immunoblotting (12% acrylamide gel). (L) The activation of autophagy (levels of LC3II, TRIB3) and apoptosis (levels of cleaved PARP) was analysed in MDA-MB-231 control and SUMO2-depleted cells. (M) Densitometry results from L as determined using ImageJ software. All experiments were performed at least three times and the data are the mean±s.e.m. of three independent experiments. Scale bar: 200 μm. *P≤0.05; n.s., not significant.

To verify that the above-described effects were based on the inhibition of SUMO1, we knocked down *SUMO1* expression in MDA-MB-231 cells by using a pool of 3 *SUMO1*-selective siRNAs. After a 72-h treatment, we found that the depletion of SUMO1 increased the accumulation of LC3 dots (reflecting the presence of autophagosomes in the cell), as well as the LC3II accumulated in these cells (Fig. 4A–D). Furthermore, we found that *SUMO1* depletion induced a clear TRIB3 upregulation in MDA-MB-231 cells (Fig. 4E,F). Similar results were obtained when we used a different pool of two *SUMO1*-selective siRNAs (Fig. S3B) or when we depleted the expression of the E2–SUMO ligase, UBC9 (Fig. 4J). Moreover, we observed an increase in cell death and apoptosis after a 5 day depletion of SUMO1 that was prevented when the last step of autophagic degradation was pharmacologically blocked with E64d and pepstatin-A (Fig. 4G–I). By contrast, no differences were observed after SUMO2/3 depletion on TRIB3 expression, autophagy or apoptosis (Fig. 4K–M).

Taken together, these findings support the idea that pharmacological or genetic inhibition of the SUMO1 pathway activates autophagy-mediated cancer cell death.

### Blockade of the SUMO1 pathway abrogates breast cancer cell invasion via inhibition of RAC1 SUMOylation

**Fig. 5.**
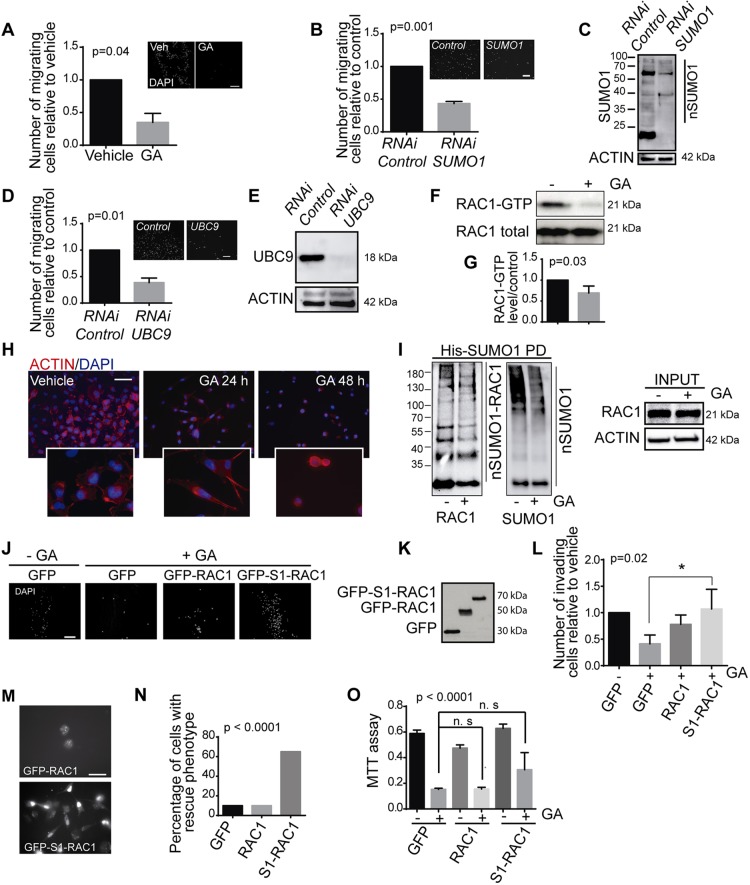
**RAC1 SUMOylation is required for cell invasion by triple negative breast cancer cells.** (A) MDA-MB-231 migration was determined in Boyden chambers. Migrating cells were determined as the number of GA-treated cells that had migrated relative to the vehicle-treated cells (set at 1) after a 24 h treatment. (B) The migration of MDA-MB-231, assessed as in A, was restricted by SUMO1 depletion. Insets in A and B show representative images of transwells. (C) Representative western blot showing SUMO1-conjugated proteins (nSUMO1) in MDA-MB-231 SUMO1-depleted cells used for migration. (D) The migration of MDA-MB-231, assessed as in A, is restricted by UBC9 depletion. (E) Representative western blot of UBC9 levels in MDA-MB-231 UBC9-depleted cells used for migration. (F) MDA-MB-231 cells were treated with GA for 24 h and RAC activation was assessed by immunoblotting. (G) The Rac-GTP bands from experiments as shown in F were quantified, and the normalized intensities were calculated relative to the controls. (H) Representative images of MDA-MB-231 cells treated with GA (10 µM) at the times indicated. (I) His–SUMO1 pulldown and RAC1–SUMO1 identification in immunoblots of HeLa cells treated with the vehicle alone or GA for 24 h. (J) Representative images of cell invasion of indicated cells as assessed in a Matrigel invasion assay. (K) Representative western blot of GFP levels for GFP-, GFP–RAC1- and GFP–SUMO1–RAC1-transfected MDA-MB-231 cells used in the invasion assay. (L) Invasion of MDA-MB-231 cells transfected with the indicated plasmids and treated with GA for 24 h. (M) Representative images of MDA-MB-231 cells transfected with wild-type GFP–RAC1 or GFP–SUMO-RAC1 after a 48 h exposure to GA. (N) Proportion of GFP–RAC1 or GFP–SUMO1–RAC1 cells expressing the constructs indicated that developed lamellipodia-membrane ruffles (‘rescue phenotype’) 48 h after GA treatment. (O) MDA-MB-231 cells were transfected with the plasmids indicated and 24 h later, they were treated with GA (10 µM) for 48 h and cell viability was determined in an MTT assay. All experiments were performed at least three times and the data are the mean±s.e.m. of three independent experiments. Scale bars: 200 μm. S1, SUMO1. *P≤0.05; n.s., not significant.

Protein SUMOylation is thought to regulate cell migration and thus, we decided to assess whether blocking the SUMO pathway might also affect the invasive capacity of cancer cells. In accordance with this hypothesis, exposure to GA under conditions that did not affect cell viability (10 µM GA for 24 h) did inhibit breast cancer cell invasion and migration by more than 50% (Fig. 5A), as did genetic depletion of SUMO1 and UBC9 (Fig. 5B–E; Fig. S4B).

The GTPase RAC1 plays a crucial role in regulating cell migration, and it has been implicated in cancer cell progression. Likewise, increased expression of this GTPase is associated with a poor prognosis in different cancer types (Mack et al., 2011). Moreover, we previously showed that RAC1 is SUMOylated in response to stimuli that trigger cell migration (Castillo-Lluva et al., 2010). When we assessed whether the inhibition of cell invasion triggered by blocking the SUMO pathway involves a dampening of RAC1 activation, we found that GA did indeed inhibit RAC1 activity (RAC1-GTP) (Fig. 5F,G). Furthermore, breast cancer cells treated with GA acquired a non-migratory phenotype and the changes in the cytoskeleton of these cells resembled those observed in cells when RAC1 signalling was abrogated. Significantly, this effect was even more pronounced after 48 h in the presence of GA (Fig. 5H).

Consequently, we analysed whether the reduction in RAC1 activity and in the invasive capacity of breast cancer cells induced by GA was dependent on the reduction in RAC1 SUMOylation. To test this hypothesis, we first confirmed that GA reduced RAC1 SUMOylation (Fig. 5I). Subsequently, to validate the functional consequences of this effect, we transfected MDA-MB-231 breast cancer cells with plasmids expressing GFP, GFP-tagged wild-type RAC1 (GFP–RAC1) or a fusion protein containing GFP and a RAC1 chimaera that mimics RAC1 SUMOylation (GFP–SUMO1–RAC1) and, therefore, cannot be de-SUMOylated or be affected by GA. We then examined the effect of exposing these cells to GA. The expression of GFP–SUMO1–RAC1 but not that of wild-type RAC1 re-established the invasive capacity of these breast cancer cells upon exposure to GA (Fig. 5J–L), as well as their migratory phenotype as assessed by the capacity to form lamellipodia and ruffled structures in the proximity of the plasma membrane (Fig. 5M,N). By contrast, expression of GFP–SUMO1–RAC1 did not affect the decrease in cell viability evoked by GA (Fig. 5O). Together, these observations support the notion that inhibiting protein SUMOylation abrogates the migratory and invasive capacity of breast cancer cell lines by inhibiting RAC1 SUMOylation.

### GA inhibits the growth of breast cancer xenografts, activates the autophagy-mediated cancer cell death pathway and inhibits RAC1 *in vivo*

**Fig. 6.**
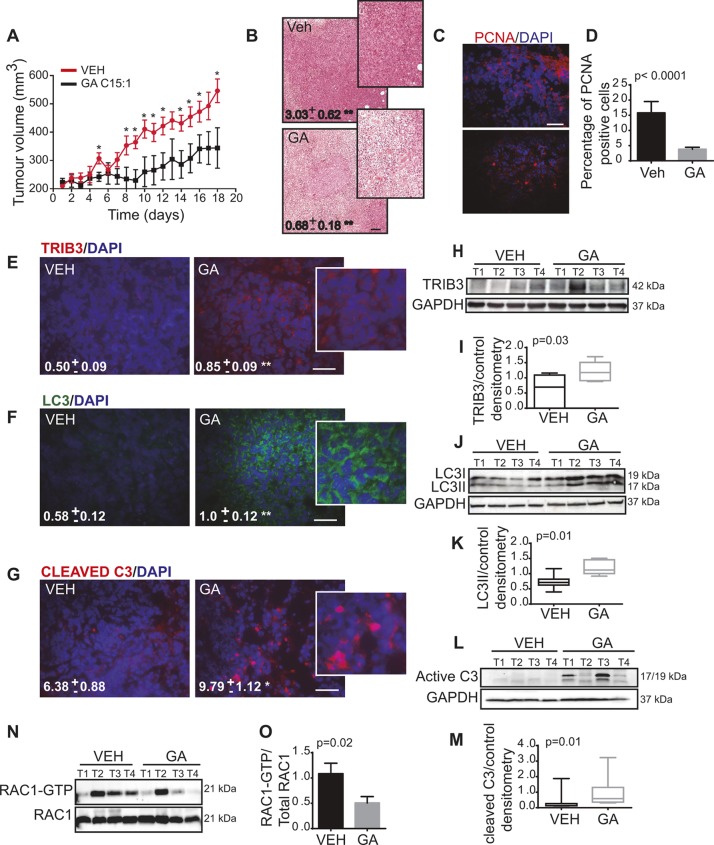
**GA inhibits the growth of subcutaneous MDA-MB-231 cell-derived xenografts.** (A) Effect of daily peritumoral injection of GA (10 mg/kg body weight) on the growth of MDA-MB-231-derived subcutaneous xenografts (mean±s.e.m., *n*=7 animals for each condition). When the tumours had reached an average volume of 200 mm^3^, they were injected peritumorally for 18 days with GA or the vehicle alone, and subjected to the following analysis. The statistical significance is indicated. *P<0.05. (B) Tumours from vehicle (VEH) and GA xenografts 18 days post-treatment (HE stain). Quantification of the intact tumour tissue relative to the necrotic and apoptotic areas performed with ImageJ software. Values inside the photomicrographs are expressed as the fraction of intact tumour tissue relative to the necrotic and apoptotic area. (C) PCNA detection in tumours treated with the vehicle alone or GA (immunofluorescence). (D) Percentage of PCNA-positive cells per tumour determined in five different treated mice. (E,F) Images of TRIB3 and LC3 levels, and quantification values obtained using ImageJ software. The values inside the photomicrographs are expressed as the TRIB3 or LC3-stained area relative to the number of nuclei in each field, and corresponding to 10 fields from three different tumours for each condition. (G) Cleaved caspase-3 (immunofluorescence), where the values inside the photomicrographs are expressed as the percentage of the cleaved caspase-3-stained cells from three different tumours for each condition. (H) Effect of GA treatment on TRIB3 expression in tumours analysed in immunoblots. (I) Densitometry quantification of results from H using ImageJ software to analyse 14 different tumours (*n*=7 vehicle tumours and *n*=7 GA-treated tumours). (J) Effect of GA on LC3 lipidation in GA-treated tumours analysed by immunoblotting. (K) Densitometry quantification of results from J using ImageJ software to analyse 14 different tumours (*n*=7 vehicle tumours and *n*=7 GA-treated tumours). (L) Effect of GA on cleaved caspase-3 in GA-treated tumours analysed by immunoblotting. (M) Densitometry quantification of results from L using ImageJ software to analyse 14 different tumours (*n*=7 vehicle tumours and *n*=7 GA-treated tumours). (N) Tumours treated with the vehicle alone or GA, assessing the activation of RAC (RAC-GTP). (O) Rac-GTP bands were quantified and the normalized intensities were calculated relative to the controls (*n*=7 tumours per group, Mann–Whitney *U*-test). The data are the means±s.d.; *P≤0.05; **P≤0.005. For I, K and M, boxes represents the 25–75th percentiles, and the median is indicated. The whiskers show the overall range. Scale bars: 200 μm.

To investigate the relevance of our findings *in vivo*, we examined the effect of administering GA to athymic mice in which a tumour xenograft had been generated by subcutaneous injection of MDA-MB-231 cells into their right flank. Treatment with GA led to a dramatic reduction in the growth of these tumours (Fig. 6A; Fig. S4C). When the histology of these tumours was evaluated following haematoxylin-eosin (HE) staining, the tumours treated with GA contained areas rich in necrotic and apoptotic tissue, with a lower mitotic index than the control xenografts (Fig. 6B). Interestingly, PCNA staining revealed that there was a significant decrease in proliferation in the tumours exposed to GA relative to the controls (Fig. 6C,D), consistent with the lower mitotic index in the HE stained tissue. Moreover, immunofluorescence and immunoblotting on the final day of treatment showed that the levels of TRIB3 (Fig. 6E,H,I), LC3II (Fig. 6F,J,K) and cleaved C3 (Fig. 6G,L,M) were enhanced in these tumours when they had been treated with GA, supporting the idea that this compound also induces autophagy-mediated cell death *in vivo*. Finally, we investigated whether blockade of the SUMO pathway with GA affected the activation of RAC1 in these MDA-MB-231 cell-derived tumours. When we performed a pulldown assay of RAC1 in extracts from vehicle and GA-treated tumours, there was less active RAC1 in the tumours treated with GA (Fig. 6N,O).

Taken together, our findings offer strong support that the mechanism by which GA inhibits the SUMO pathway, and negatively regulates tumour survival and invasion *in vitro*, also operates *in vivo*.

## DISCUSSION

Normal tissues control the production and release of signalling molecules in order to maintain their homeostasis and architecture. The dysregulation of such signals favours tumour progression, and the proliferation, survival and dissemination of cancer cells (Hanahan and Weinberg, 2011). SUMOylation is a PTM that alters the activity of proteins involved in different cellular processes and, thus, SUMOylation can regulate various biological processes (Johnson, 2004). Indeed, alterations in the SUMOylation–deSUMOylation equilibrium have been associated with various diseases, including cardiac pathologies, neurodegeneration and cancer (Yang et al., 2017). Here, we explored the importance of the SUMO1 pathway in controlling tumorigenesis by inhibiting SUMO1 expression genetically and pharmacologically. Our findings demonstrate that SUMO1 inhibition negatively regulates the tumorigenic properties of cancer cells via two different mechanisms: (1) stimulation of autophagy-mediated cancer cell death, and (2) inhibition of cancer cell invasiveness via regulation of RAC1 SUMOylation.

In our work, we used two pharmacological inhibitors of protein SUMOylation acting through different mechanisms: C15:1 GA and 2D-08. GA is a phenolic acid obtained from leaves, nuts and the external seed coat of *G. biloba*, which exert several pharmacological effects including inhibition of tumour growth in animal models of cancer (Baek et al., 2017; Li et al., 2017; Liu et al., 2018b). Specifically, we used the C15:1 monomer that inhibits protein SUMOylation by blocking the E1–SUMO1 interaction (Fukuda et al., 2009). We show that, as with other molecular species (Li et al., 2017; Liu et al., 2018a,b), the viability of breast and prostate cancer cells was compromised by GA in a dose-dependent manner. However, exposure to GA (10 µM) for 24 h impaired the formation of the SUMO1 complexes without affecting cell viability. Thus, in these conditions, we could investigate the implication of SUMO inhibition in tumorigenesis.

In our study, we also used the SUMO E2 inhibitor 2-D08, which has been shown to block SUMOylation (Choi et al., 2018; Kim et al., 2014), although GA proved to be a more potent SUMO inhibitor than 2-D08, producing a 40% reduction in SUMO1-complexes after 24 h while 2-D08 failed to reduce SUMOylation in that time. However, after a 48 h exposure to 2-D08, similar inhibition of SUMOylation was evident as that produced by GA and a similar degree of autophagy-induced cell death was produced by both agents.

Findings presented in this work demonstrate by using different experimental approaches that SUMO1 inhibition induces autophagy-mediated cancer cell death. Thus, inhibition of SUMO1 induces the upregulation of several autophagy related genes such as *ATG1*, *ATG5*, *ATG7*, *BECN1* or *AMBRA1* at early time points. Moreover, the genetic depletion of some of this key autophagy regulatory genes (or the pharmacological inhibition of autophagy, E64d and pepstatin-A) prevents SUMO1 blockade-induced apoptosis and cell death. It is worth noting that, unlike genetic inhibition of SUMO1, the depletion of SUMO2/3 does not induce autophagy-mediated cell death, suggesting that the effects observed in this work take place specifically through the blockade of SUMO1 conjugation.

GA and 2D-08 has been shown to inhibit the SUMO pathways acting on the E1–SUMO and E2–SUMO enzymes respectively. However, whether these compounds, and specifically GA, may act on other targets apart from these SUMO-regulatory enzymes is unknown. For example, different studies have shown that treatment with GA inhibits lipogenesis (Ma et al., 2015), and the EGF (Li et al., 2017) and TGF-β (Baek et al., 2017) signalling pathways, among others. However, none of these studies investigated whether the changes observed on those signalling pathways relied on the modulation of protein SUMOylation. Our results clearly demonstrate that treatment with GA and 2-D08 inhibits protein SUMOylation in the cellular models employed in this study; however, it cannot be ruled out that GA (or 2-D08) may produce their effects at least in part acting independently of the regulation of protein SUMOylation.

TRIB3 is a member of the Tribbles family of pseudokinases that has been implicated in autophagy-mediated cancer cell death after administration of different anti-cancer agents (Salazar et al., 2009a,b). In our work, we demonstrate that pharmacological or genetic inhibition of the SUMO pathway induces autophagy, apoptosis and cell death in a TRIB3-dependent manner. Moreover, we found that TRIB3, as well as other markers of the autophagy-mediated cell death pathway, are upregulated *in vivo* upon GA administration to mice bearing breast cancer xenografts. However, TRIB3 depletion partially rescued the decrease in MDA-MB-231 viability evoked by GA treatment, indicating that there could be additional (TRIB3-independent) mechanisms by which GA regulates cancer cell death.

RAC1 plays an important role in controlling the cytoskeletal reorganization associated with cell motility. In a cancer context, the enhanced activation of this protein has been implicated in metastasis and invasiveness. Furthermore, RAC1 is SUMOylated by SUMO1 and this modification is required to maintain the activity of the protein during cell migration (Castillo-Lluva et al., 2010). Here, we demonstrate that RAC1 SUMOylation is important for optimal breast tumour cell migration and invasion but not for other RAC1 functions, such as proliferation and survival. GA inhibits the migration of various tumour cell types (Baek et al., 2017; Hamdoun and Efferth, 2017; Yang et al., 2014), and we show that this effect on cell invasion is related to the inhibition of SUMO conjugation.

Overall, the data presented here enhance our understanding of the mechanisms by which protein SUMOylation regulates cancer cell biology. Specifically, we found that inhibiting protein SUMOylation induces autophagic cell death through the upregulation of the pseudokinase TRIB3, and it impairs cancer cell invasiveness by inhibiting activation of the small GTPase RAC1. Blocking the SUMO pathway would reduce RAC1 SUMOylation, thereby diminishing the amount of active RAC1 available to drive the cell migration and invasion programme. Nevertheless, further studies will be required to fully understand the mechanisms involved in the induction of autophagy following the inhibition of SUMOylation. In any case, our findings do support the notion that blocking protein SUMOylation can be explored as a potential therapeutic strategy to fight cancer.

## MATERIALS AND METHODS

### Reagents

Ginkgolic acid C15:1 (#02580585), 2′,3′,4′-trihydroxy-flavone, 2-(2,3,4-trihydroxyphenyl)-4H-1-Benzopyran-4-one (2-D08; #SML1052), 3-(4,5-dimethylthiazol-2-yl)-2,5-diphenyltetrazolium bromide (MTT; #M5655), Crystal Violet (#C3886), and the E64d (10 µmol/l; #E8640) and Pepstatin-A (10 µg/ml; #P5318) inhibitors were all purchased from Sigma-Aldrich (St Louis, MO). HeLa cells were transfected using LT1 (Mirus Bio, Madison WI; #MIR 2304) and MDA-MB-231 cells with Lipofectamine 2000 (Thermo Fisher Scientific, Waltham, MA; #11668027). The *SUMO1* siRNA (#sc-29498), *UBC9* siRNA (#sc-36773), SUMO2/3 siRNA (#sc-37167), *BECLIN1* siRNA (sc-29797), *AMBRA1* siRNA (sc-96257) and *TRIB3* shRNA lentiviral particles (#sc-44426-V) were obtained from Santa Cruz Biotechnology (Heidelberg, Germany). *SUMO1* siRNA #2 was from Sigma-Aldrich (#EHU106621). For siRNA transfection, DharmaFECT 2001 was used (Dharmacon, Lafayette, CO; #T2001).

### Protein analyses

Proteins were analysed in western blots as described previously (Castillo-Lluva et al., 2015). In brief, proteins were extracted from tumours frozen in RIPA buffer [150 mM NaCl, 1% (v/v) NP40, 50 mM Tris-HCl pH 8.0, 0.1% (v/v) SDS, 1 mM EDTA, 0.5% (w/v) deoxycholate], while proteins from cell lines were extracted in TNES buffer [100 mM NaCl, 1% (v/v) NP40, 50 mM Tris-HCl pH 7.6, 20 mM EDTA] containing protease and phosphatase inhibitor cocktails (Sigma-Aldrich; #P8340). The proteins recovered were quantified using the BCA Protein Assay Kit (Thermo Fisher Scientific; #23228), resolved by SDS-PAGE on 4–15% gradient gels (purchased from Bio-Rad, Berkeley, CA; #456-8085) and transferred to polyvinylidene difluoride membranes (Immobilon-P, Millipore, Burlington, MA). The membranes were then probed with the following primary antibodies: anti-cJUN (1:500; H72, Santa Cruz Biotechnology; #sc-1694), anti-Bax (1:1000; Cell Signaling Technology; #2772S), anti-TRIB3 (1:1000; Abcam, Cambridge, UK; #ab75846), anti-tubulin (1:1000; DM1A, Sigma-Aldrich; #T6199), anti-RAC (1:1000; clone 102; BD Biosciences, Franklin Lakes, NJ), anti-LC3 (1:1000, Sigma-Aldrich; #L7543), anti-cleaved caspase-3 (1:1000, Cell Signaling Technology; #9662), anti-GAPDH (1:1000, Sigma-Aldrich; #G8795), anti-cleaved PARP (1:1000, Cell Signaling Technology; #D214), anti-UBC9 (1:1000, Abcam; #ab75854), anti-SUMO1 and anti-SUMO2/3 (provided by Ron Hay's laboratory, School of Life Sciences, University of Dundee, Dundee, UK), anti-GFP (Sigma-Aldrich; #SAB1305545), and anti-ATG5 (Cell Signaling Technology; #12994S). Antibody binding was detected with horseradish peroxidase (HRP)-conjugated anti-mouse, anti-rabbit or anti-sheep secondary antibodies (1:10,000 dilution, Bio-Rad), and visualized by enhanced chemiluminescence (Bio-Rad). The images were obtained with the ImageQuant LAS 500 chemiluminescence CCD camera (GE Healthcare Life Sciences, Chicago, Illinois, USA).

### Cell culture

MDA-MB-231 and MCF7 breast cancer cells (ATCC) were grown in complete DMEM, containing 4.5 g/l glucose and L-glutamine (Sigma-Aldrich). LnCaP and 22Rv1 prostate cancer cells, and BT474 breast cancer cells (ATCC), were grown in RPMI medium (Sigma-Aldrich). RasV^12^/T-large antigen-transformed ATG5^+/+^ and ATG5^−/−^ cells (Salazar et al., 2009b) were grown in in DMEM. In all cases, the medium was supplemented with 56 IU/ml penicillin, 56 mg/l streptomycin (Invitrogen, Carlsbad, CA) and 10% fetal bovine serum (FBS, LINUS #16sV30180.03), and the cells were maintained at 37°C in a humid atmosphere containing 5% CO_2_. All cells were routinely tested for mycoplasma contamination. Cell lines were authenticated at the Genomics Core Facility (Instituto de Investigaciones Biomédicas ‘Alberto Sols’ CSIC-UAM, Madrid, Spain) using the STR PROFILE DATA, STR amplification kit, GenePrintR 10 System (Promega), STR profile analysis software GeneMapper^®^ v3.7 (Life Technologies) and the Genomic Analyzer System ABI 3130 XL (Applied Biosystems). Cell viability was determined in MTT assays following the manufacturer's instructions (Sigma-Aldrich; #M5655). For transfection experiments with SUMO1 and control siRNA, 10^6^ cells were transfected using the DharmaFECT 1 Transfection Reagent (Dharmacon; #T-2001) and incubated for up to 72 h before setting up the experiment. Infection with TRIB3 shRNA human lentiviral particles was performed by using a pool of concentrated transduction-ready viral particles containing three target specific shRNAs (or three non-targeted control shRNA) constructs (Santa Cruz Biotechnology). After the infection of human cancer cell lines, stably silenced (or control shRNA transduced) cells were selected.

### RAC1 activation and the PAK-PBD pulldown assay

RAC1 activation was determined using a RAC1 activation pulldown assay (Thermo Fisher Scientific; #16118) following the manufacturer's instructions. Cells were treated with 10 µM of GA C15:1 for 24 h before RAC1 activity was determined.

### Boyden chamber cell migration/invasion assay

Cell migration was assayed in Boyden chambers (8.0 μm pore-size polyethylene terephthalate membrane with a cell-culture insert: VWR, Radnor, PA; #VWRI734-2744). The cells were trypsinized and counted, and cell suspensions containing 5×10^4^–10×10^4^ cells in 300 μl of serum-free medium were added to the upper chamber, with 500 μl of the appropriate medium added to the lower chamber. The transwell inserts were incubated for 24 h at 37°C, and the cells on the inside of the transwell inserts were removed with a cotton swab, whereas those on the underside of the insert were fixed and stained. Photographs were taken of five random fields and the cells were counted to calculate the proportion of transmigrated cells. Boyden chambers with Matrigel (Cell Biolabs, San Diego, CA; #CBA-110) were used to assay cell invasion.

### Immunofluorescence microscopy

MDA-MB-231 cells were plated and grown on glass coverslips for 24 h. The cells were fixed for 10 min in PBS with 4% paraformaldehyde at room temperature and the coverslips were then stored in PBS with 0.05% azide at 4°C until the cells were immunostained. Briefly, the cells were permeabilized by incubating for 3 min at 4°C in PBS containing 0.5% Triton X-100 and after that, the coverslips were incubated overnight at 4°C with a 1:500 dilution of the primary antibodies against Ki67 (Thermo Fisher Scientific, #PA1-21520), LC3 (Sigma-Aldrich; #L7543) or 1:50 of cleaved caspase-3 (Asp175; Cell Signaling; #9661). After rinsing thoroughly, the coverslips were incubated with Alexa Fluor 488 or 594 goat anti-mouse-IgG or anti-rabbit-IgG secondary antibodies (Invitrogen), counterstained with DAPI for 30 s, and then mounted on glass slides with DAPI using the Gold antifade reagent (Invitrogen; #P36935). Immunofluorescence was analysed under a Zeiss microscope (Zeiss Axioplan 2).

### Apoptosis assays

MDA-MB-231 apoptotic cells were quantified using an Annexin V-fluorescein isothiocyanate (FITC)/propidium iodide (PI) apoptosis detection kit (Sigma-Aldrich; #APOAF). Cells were seeded at a density of 10^6^ cells in 10 cm dishes. After treatment with 10 µM of GA for 48 h in serum-free medium, cells were harvested and Annexin-V-FITC/PI labelling was performed according to the manufacturer's instructions. The stained cells were analysed with a flow cytometer (FACSCalibur, BD Biosciences).

### Nickel affinity purification

His6–SUMO–1-binding proteins were purified from cell lysates using nickel-nitrilotriacetic acid agarose (QIAGEN, Hilden, Germany; #30230), as described previously (Castillo-Lluva et al., 2010).

### Xenografts

Tumours were generated in nude mice (Athymic nude-Foxn1 6 weeks old, ENVIGO RMS, Barcelona, Spain) by subcutaneous injection of 10×10^6^ MDA-MB-231 cells in PBS supplemented with 0.1% glucose. When the tumours had reached an average volume of 200 mm^3^, the animals were randomly assigned to different treatment groups and injected peritumourally for 18 days with GA (10 mg/kg body weight per day) or the vehicle alone (ethanol) in 100 µl of PBS, supplemented with 5 mg/ml defatted and dialyzed BSA. Tumour growth was determined with digital callipers and the tumour volume was estimated on each day using the formula: tumour volume=length×width^2^×0.5. We obtained the tumour growth rate after transforming the data logarithmically, and we estimated a linear regression curve for each tumour, evaluating the means of the slopes of these lines for each group.

This research was carried out in accordance with the regulatory ethical standards, and in compliance with national and international guidelines, and it was approved by the authors' institutional review board.

### Statistical analyses

Unless otherwise indicated, the data are expressed as the mean±standard error of the mean (s.e.m.), and evaluated with the Mann–Whitney *U*-test or *t*-test for two groups, with the Kruskal–Wallis test (followed by Dunn's multiple comparison post-test) or by ANOVA (followed by Turkey's multiple comparisons test) for more than two groups. The percentage of positive cells was evaluated using the χ-squared test and the tumour volumes in the mice were compared using a multiple *t*-test. For all the analyses, *P*-values ≤0.05 were considered statistically significant.

## Supplementary Material

Supplementary information
