# Supplementary material for: Inhibiting SUMO1-mediated SUMOylation induces autophagy-mediated cancer cell death and reduces tumour cell invasion via RAC1
Source: J Cell Sci. 2019 Oct 22;132(20):jcs234120. doi: 10.1242/jcs.234120 (PMC6826015; doi:10.1242/jcs.234120)
Supplement: Supplementary information [file joces-132-234120-s1.pdf]

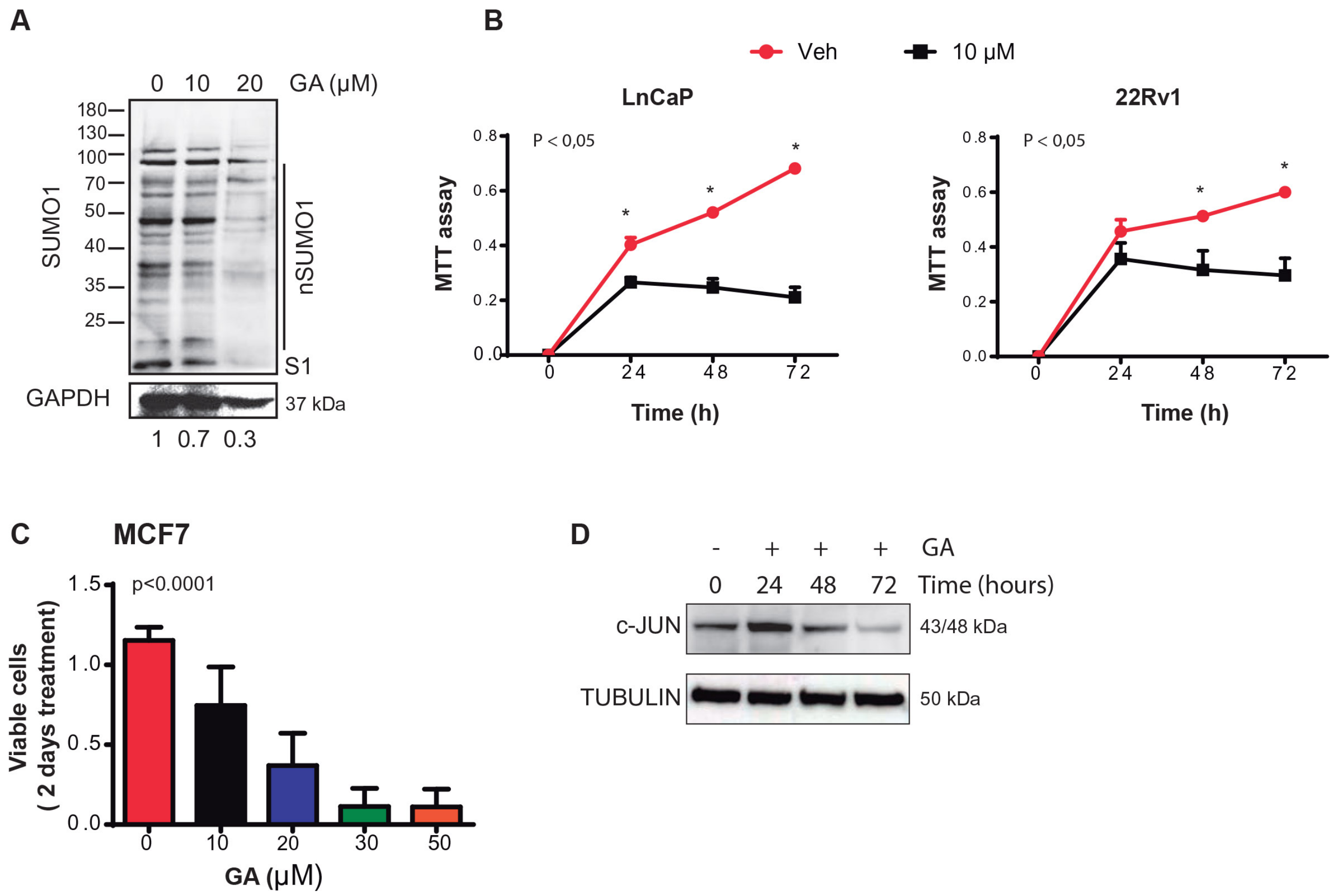

Figure S1.

(A) SUMO conjugated protein expression (nSUMO1) in MDA-MB-231 cells treated with GA at various concentrations for 24 hours and analyzed by immunoblots (4-15% gradient acrylamide gel). (**B**) Prostate cancer cell lines (LnCap and 22Rv1) were treated with GA and their viability was assessed in a crystal violate assay.(**C**) Viability of MCF7 cells after treatment with different concentrations of GA for two days. (**D**)The c-JUN protein in MDA-MB-231 cells after treatment with GA at various concentrations.

Journal of Cell Science • Supplementary information

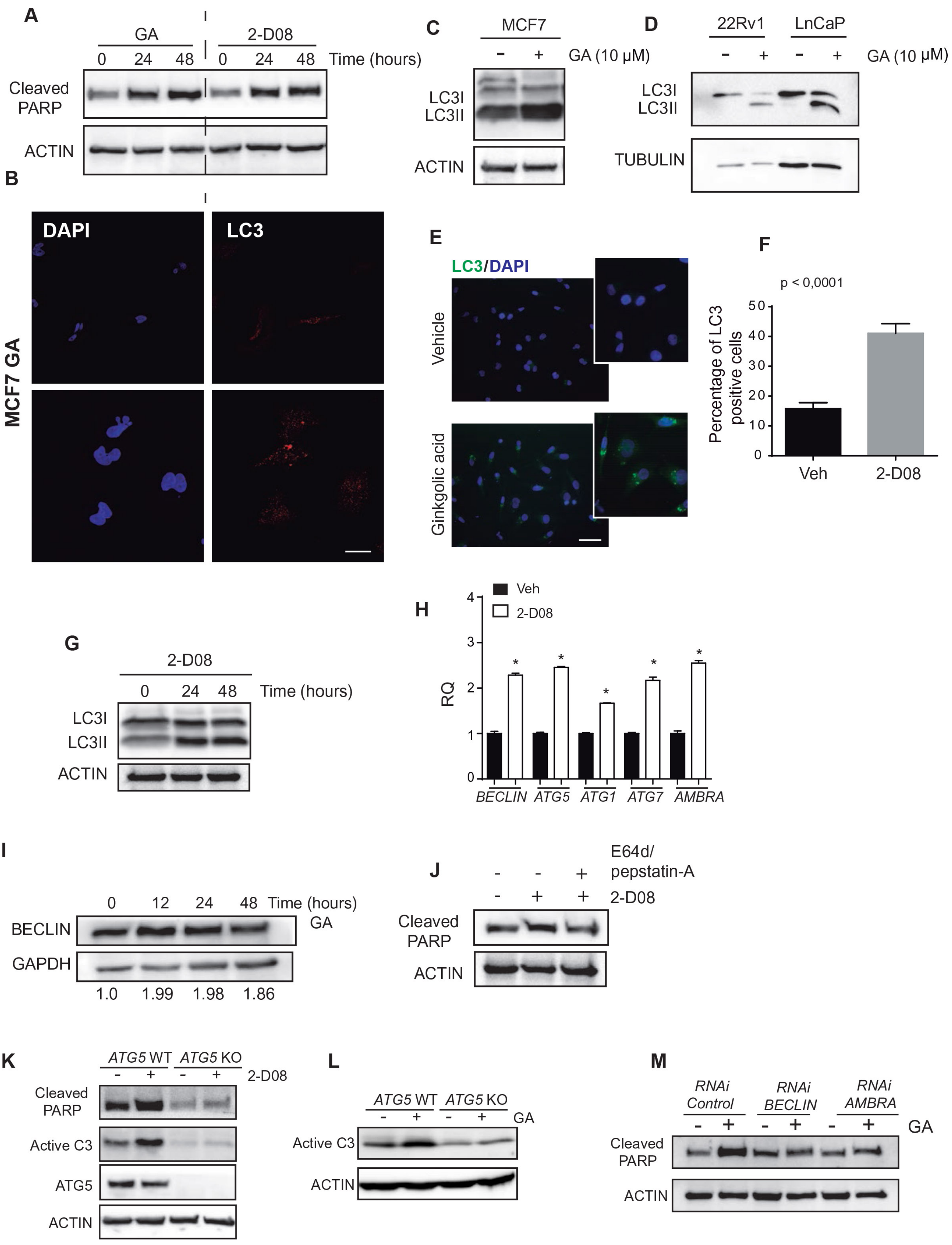
A
GA
2-D08
0 24 48 0 24 48 Time (hours)
Cleaved PARP
ACTIN
B
DAPI
LC3
MCF7 GA
C
MCF7
- + GA (10 μM)
LC3I
LC3II
ACTIN
D
22Rv1
LnCaP
- + - + GA (10 μM)
LC3I
LC3II
TUBULIN
E
LC3/DAPI
Vehicle
Ginkgolic acid
F
p < 0,0001
Percentage of LC3 positive cells
50
40
30
20
10
0
Veh
2-D08
G
2-D08
0 24 48 Time (hours)
LC3I
LC3II
ACTIN
H
Veh
2-D08
RQ
4
3
2
1
0
BECLIN ATG5 ATG1 ATG7 AMBRA
I
0 12 24 48 Time (hours)
BECLIN
GAPDH
GA
1.0 1.99 1.98 1.86
J
E64d/ pepstatin-A
2-D08
Cleaved PARP
ACTIN
K
ATG5 WT
ATG5 KO
2-D08
Cleaved PARP
Active C3
ATG5
ACTIN
L
ATG5 WT
ATG5 KO
GA
Active C3
ACTIN
M
RNAi Control
RNAi BECLIN
RNAi AMBRA
GA
Cleaved PARP
ACTIN

Fig. S2

(**A**) Activation of apoptosis by cleaved PARP was assessed in MDA-MB-231 treated with GA (10 µM) or 2-D08 (20 µM) (**B**) Representative image of LC3 immunostaining of MCF7 (**C**) Effect of GA (10 µM) on LC3 in MCF7 (**D**) Effect of GA on LC3 lipidation in prostate cancer cells. (**E**) Representative image of LC3 immunostaining of MDA-MB-231 treated for 24 hours with 20 µM of 2-D08. (**F**) The percentage of cells with LC3 dots relative to the total number of cells after 24 h treatment (Student´s t-test). (**G**) Effect of 2-D08 on LC3 lipidation in MDA-MB-231 breast cancer cell. (**H**) Effect of 2-D08 on autophagy expression markers by qPCR. (**I**) The effect of GA on BECLIN expression by immunoblots (**J**) Activation of apoptosis by cleaved PARP was assessed in MDA-MB-231 treated with 2-D08 in the presence of E64d and Pepstatin-A. (**K**) Activation of apoptosis by cleaved PARP and cleaved C3 was assessed in MEF *ATG5KO* cells in the presence of 2-D08 at 48 hours. (**L**) Activation of apoptosis by cleaved C3 was assessed in MEF *ATG5KO* cells in the presence of GA at 48 hours. (**M**) Activation of apoptosis by cleaved PARP was assessed in MDA-MB-231 *BECLIN* and *AMBRA* depleted cells in the presence of GA.

Journal of Cell Science • Supplementary information

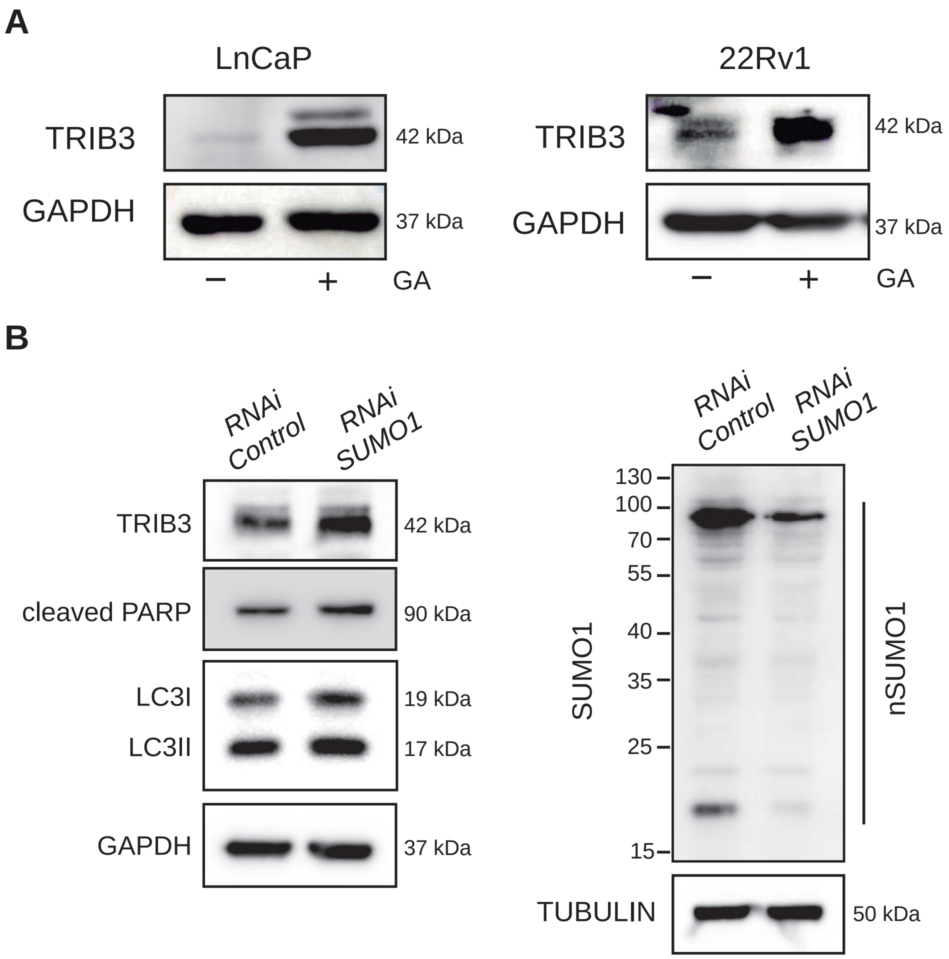

Fig. S3

(**A**) The TRIB3 protein in prostate cancer cells treated with GA (10 µM) was analysed by immunoblots. (**B**) MDA-MB-231 control and SUMO1 depleted cells were analysed for activation of autophagy (LC3II, TRIB3) and apoptosis by cleaved PARP and for SUMO conjugated proteins in immunoblots.

Journal of Cell Science • Supplementary information

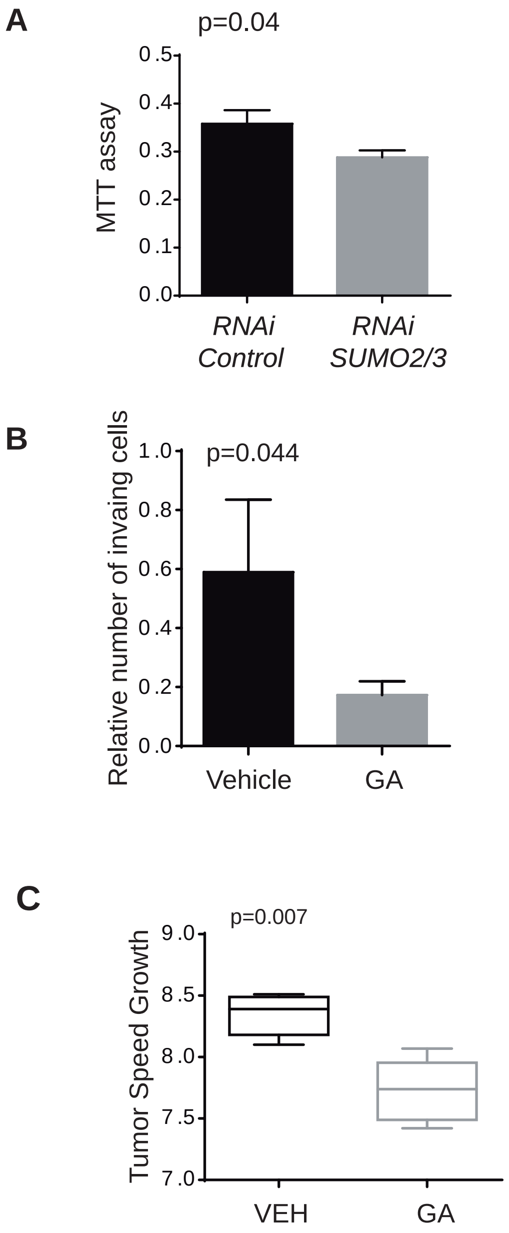

Fig. S4

(**A**) The viability of MDA-MB-231 SUMO2/3 depleted cells was evaluated with a MTT assay at 72 hours post-depletion. (**B**) MDA-MB-231 cells were treated with GA and cell invasion was assayed in Boyden chambers. (**C**) Comparison of the growth rate of tumours treated with GA or vehicle.

Journal of Cell Science • Supplementary information
